# Teacher support, grit and L2 willingness to communicate: the mediating effect of foreign language enjoyment

**DOI:** 10.1186/s40359-024-01877-5

**Published:** 2024-07-09

**Authors:** Yanyu Yang, Yongze Cui, Suhua Yao

**Affiliations:** 1 Department of Public Courses, Guangdong Police College, No. 500 Binjiangdong Road, Haizhu District, Guangzhou, 510230 Guangdong Province China; 2 School of Economics and Management, Shanxi Normal University, No. 339 Taiyu Road, Xiaodian District, Taiyuan, 030031 Shanxi Province China; 3 Center for Linguistics and Applied Linguistics, Guangdong University of Foreign Studies, No. 2 Baiyun Road, Baiyun District, Guangzhou, 510420 Guangdong Province China; 4 Basic Teaching Department, Guangdong Comminication Polytechinc, No. 789 Tianyuan Road, Tianhe District, Guangzhou, 510650 China

**Keywords:** Teacher support, Grit, Foreign language enjoyment, L2 willingness to communicate

## Abstract

Informed by some relevant theories of positive psychology, the current study examined the interrelations among teacher support, grit, and L2 willingness to communicate (WTC), focusing mainly on the mediating role of foreign language enjoyment (FLE). A sample of 619 university students in China participated in this cross-sectional survey. Structural equation modeling (SEM) was used to analyze the gathered data. The results showed that grit could directly and positively predict L2 WTC. Furthermore, teacher support and grit could affect L2 WTC via the mediating role of FLE. These findings served as empirical evidence from the second language acquisition (SLA) domain for positive psychology, revealing the influential mechanism shaping the interconnectedness among all the constructs. The study concluded with a discussion of pedagogical implications and suggestions for future research.

## Introduction

**Fig. 1:**
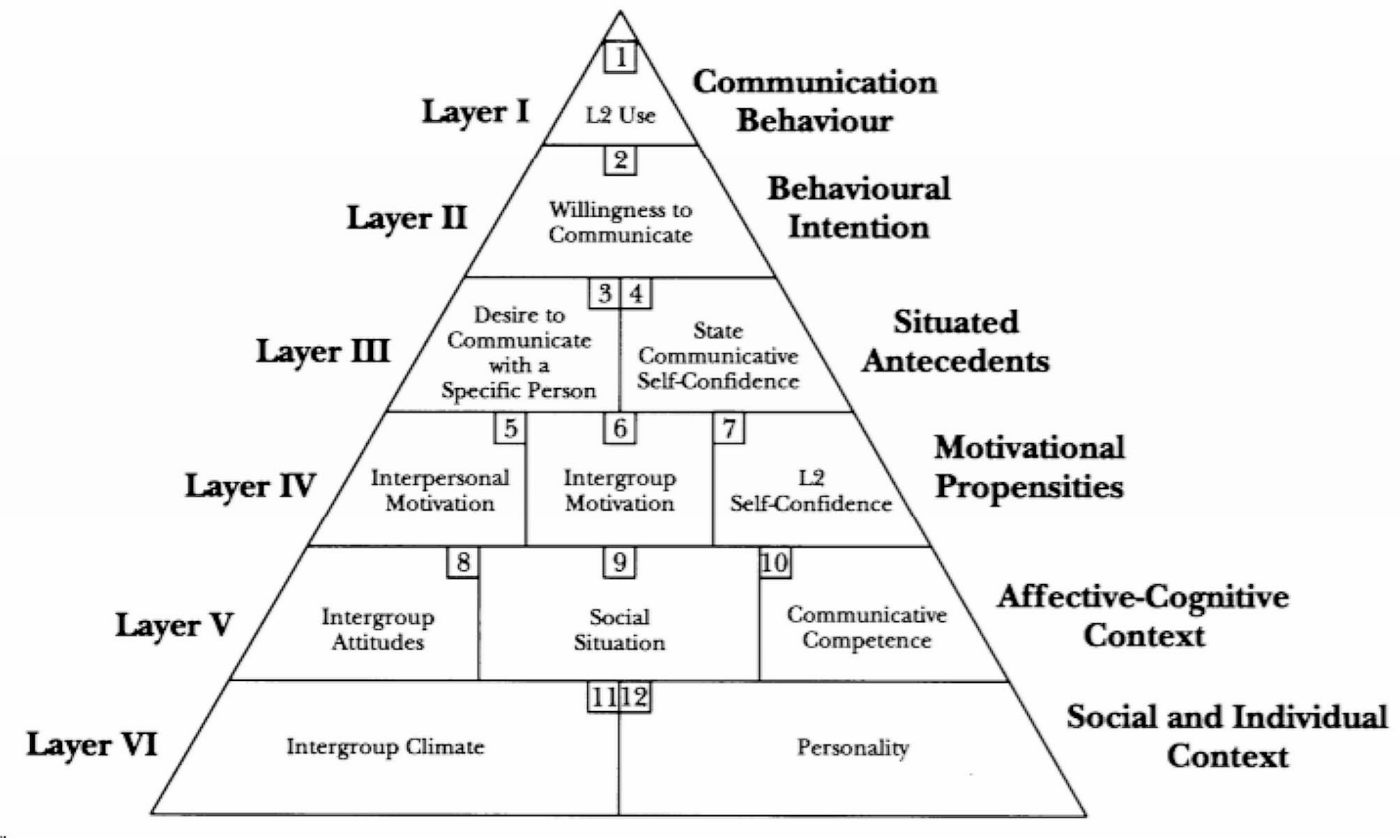
The pyramid model of WTC (MacIntyre et al., 1998, p. 547)

For many years, second and foreign language (L2) instruction in Asian countries has been dominated by grammar-translation or rote-learning methods in highly structured classrooms [1, 2]. In recent decades, with the introduction of communicative approaches to language teaching worldwide, communication has become the main purpose of L2 education and a tool for facilitating language acquisition [3, 4]. Being willing to communicate is also an integral part of becoming a fluent L2 speaker and the ultimate goal in learning [5, 6]. However, as demonstrated in many case studies, L2 learners have distinct levels of willingness to communicate [7, 8]. Some may seize every opportunity to speak up and practice their language skills, while others have some avoidance tendencies and remain silent in and out of class [9]. Generally, higher levels of L2 willingness to communicate (WTC) are associated with more frequent language use, which may eventually contribute to communicative proficiency [10]. Therefore, language researchers are interested in probing into its determinants and finding out possible ways to improve learners’ L2 WTC. Based on the pyramid model of WTC (see Fig. 1), various psychological determinants ranging from intrapersonal and interpersonal to situational factors may sway speakers’ decisions to either engage in communication or desist [6]. Unfortunately, previous studies have focused mainly on investigating superficial bivariate relationships, such as the relationship between teacher support and L2 WTC, as well as that between grit and L2 WTC [11, 12]. There is a dearth of studies that explore how institutions and personality factors jointly influence L2 WTC and the potential role of emotions as a mediator. Seligman and Csikszentmihalyi suggested that the Three Pillars of positive psychology, namely positive institutions, positive personality traits, and positive subjective experience, would help individuals flourish [13]. In the field of SLA, these Three Pillars are intricately tied to one another, and their interplay is conducive to language development [14]. Supported by the above theory, an inference is drawn that L2 learners with a higher level of grit may have a more positive perception of teacher support, and L2 learners who receive more frequent teacher support are also more likely to be grittier and experience more positive emotions, which may ultimately promote their L2 studies, including L2 WTC. Although all the aforementioned constructs have been discussed separately to a greater or lesser extent in the fields of educational psychology, positive psychology, or SLA [15–23], very few studies, to the best of our knowledge, have endeavored to delve into the complex associations among them in question. Thus, from a positive psychology perspective, this study seeks to bridge the existing gap by examining whether positive institutions (e.g., teacher support), positive personality traits (e.g., grit), and positive emotions (e.g., FLE) are related to L2 learners’ WTC and furthermore, how the mediating role of FLE is realized in the relationships between teacher support, grit, and L2 WTC. We hope that our research findings will confirm the contributions of positive psychology to English as a foreign language (EFL) pedagogy, yield implications for EFL teaching, suggest recommendations for enhancing students’ L2 WTC, and offer a point of departure for future studies.

## Literature review

### L2 willingness to communicate (WTC)

WTC in L2 settings is conceptualized as “a readiness to enter into discourse at a particular time with a specific person” [6]. The factors shaping WTC can be vividly depicted in a multilayered pyramid model (see Fig. 1) encompassing both stable (layers IV, V, and VI) and changing contextual variables (layers I, II, and III). A growing body of empirical research has been conducted to explore variables in this model that influence WTC in L2 contexts, including classroom environment, personality, motivation, L2 competence, and emotions [8, 22–25]. For example, in a large-scale questionnaire survey administered by Li et al. among 2268 university students in China, it was found that classroom environment had a direct effect on students’ L2 WTC and three L2 emotions mediated the link between classroom environment and L2 WTC [22]. Oz carried out a study involving 168 university students in Turkey to test the influence of the Big Five personality traits on L2 WTC. The results indicated that extraversion, agreeableness, and openness to experience were strongly associated with L2 WTC, while no associations were found between neuroticism, conscientiousness, and L2 WTC [24]. Another line of study performed by Chen et al. among 273 Taiwanese university students demonstrated that learners’ intrinsic motivation was one key factor promoting their L2 WTC in the intercultural background [23].

**Fig. 2:**
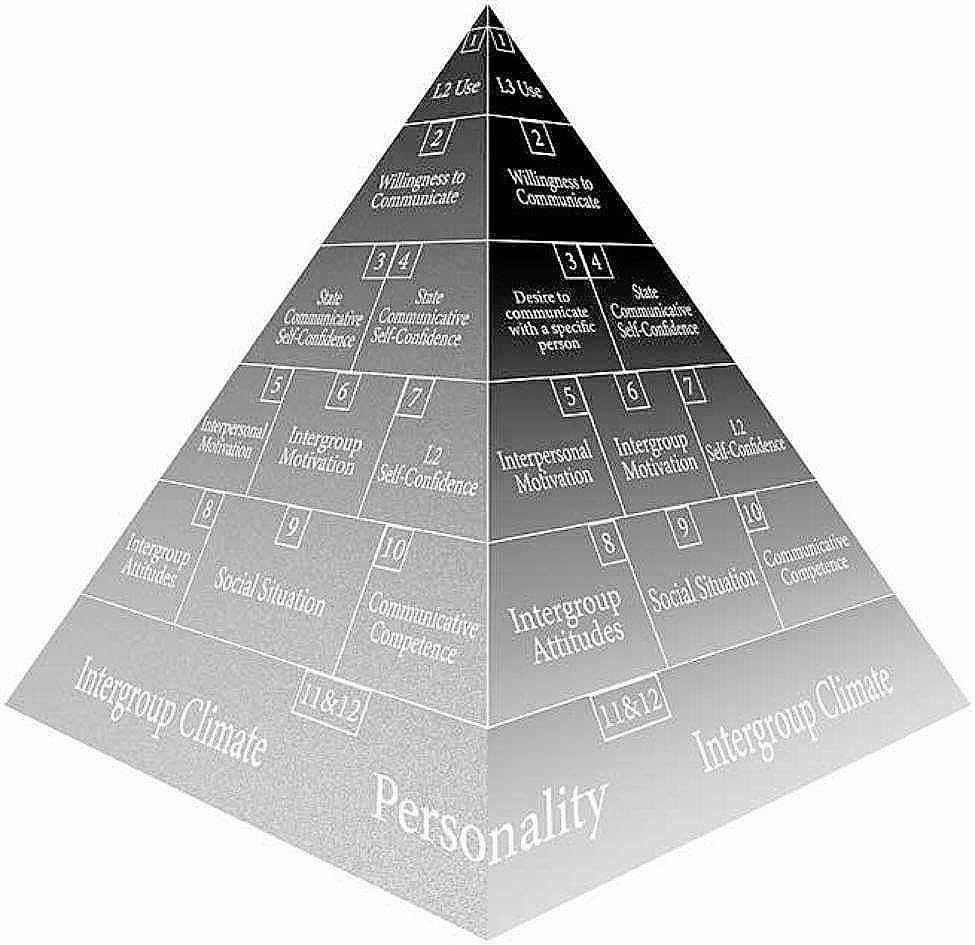
A 3D model of the WTC pyramid (Henry & MacIntyre, 2023, p. 262)

Although most previous studies regarding WTC were based on a quantitative approach, it is worth noting that in recent years, some mixed methods studies and qualitative approaches have emerged, aiming to reveal its dynamic nature. For example, in a mixed-method study involving 52 intermediate Spanish learners, Jaramillo Cherrez and Nadolny discovered that students’ WTC can be significantly enhanced by video discussion tasks in the Flip Group [26]. In a newly published book, Henry and MacIntyre unveiled the communication behaviors and language choices of some adult migrants residing in a completely new cultural/linguistic context using an in-depth qualitative longitudinal approach [27]. Specifically, the dynamic nature of WTC in multilingual contexts was demonstrated by a revised 3D pyramid model (see Fig. 2), enlightening processes that influence communication, migration, and well-being [27]. In this 3D model, factors at the bottom (i.e., intergroup climate and personality) still remain unchanged. However, as we move upward, factors at each ascending level are more prone to fluctuate when a code switch occurs, which can be recognized as evidence reflecting changes occurring in the factors underpinning the construction of WTC over a more extended period [27].

### Teacher support and L2 WTC

Teachers play a crucial role in providing different forms of support, such as academic support, instrumental support, and emotional support to students. Academic support involves not only specific knowledge imparted by teachers but also the feedback they offer in accordance with students’ performance [28]. Instrumental support refers to instrumental resources and practical help provided by teachers to assist students in developing their academic skills. Emotional support means that teachers are concerned about students’ physical and emotional well-being [29]. As support provided by teachers is a critical dimension of the classroom social environment [30], the latter falling into the category of “institutions”, we thereby consider teacher support as one construct of the Three Pillars (specifically “positive institutions”) for further discussion. A review of the literature indicates that teacher support can significantly affect learners’ motivation, creative thinking, academic engagement, social competence, and academic achievement [15, 31–34]. For instance, a questionnaire survey conducted by Sadoughi and Hejazi with the participation of 435 EFL freshmen from 5 Iranian universities verified that teacher support could both directly and indirectly predict student academic engagement [15]. Chiu et al. found that teacher support played a pivotal role in elevating student motivation to learn with AI technologies [31]. Drawing on 71 empirical articles, Tao et al. utilized a meta-analytic approach and revealed that teacher support exerted the most substantial influence on academic achievement and course grades among upper-secondary students [34].

Prior research has also underlined the role of teachers in impacting students’ L2 WTC. For example, Zarrinabadi invited 97 undergraduates to describe the situations in which teachers influenced their L2 WTC in English orally via a qualitative method. As a result, teachers’ error correction, decisions on the topic, and support impinge on students’ L2 WTC [11]. The extent to which teacher support affects L2 WTC, however, is still underexplored in academia. In a recent attempt, Hejazi et al. recruited 551 Iranian EFL learners from 12 private language schools in Teran for a questionnaire survey. The results uncovered that teacher support was directly and positively linked to L2 WTC [16]. However, their relationship was significant only among learners with medium/high levels of growth language mindset. Another study performed by Peng and Woodrow reported different results. According to their research, teacher support, as one dimension of the classroom environment, was found to significantly and positively predict L2 WTC [35]. It can be seen from the incongruent literature above that more empirical research is needed to provide an in-depth understanding of the intercorrelation between teacher support and L2 WTC.

### Grit and L2 WTC

As a non-cognitive higher-order personality trait, grit refers to “perseverance and passion for long-term goals” and has two facets: perseverance of effort (POE) and consistency of interest (COI) [36]. During the previous two decades, girt has gained widespread popularity in educational psychology research, and in recent years, it has begun to attract the attention of SLA researchers because L2 learning is beset with obstacles and hurdles [37]. The relevant literature discloses that some studies have suggested a higher-order two-factor structure. In contrast, others have argued for a lower-order one-factor structure regarding the factor structure of grit [19]. Additionally, there is a paucity of research on the relationship between grit and academic achievement [17–19, 38, 39]. Their findings, however, were inconsistent in different learning contexts. For example, Zhao and Wang reported that both POE and COI were significant predictors of language achievement among Chinese EFL learners [18]. In a questionnaire survey among 226 intermediate English language learners in Iran, Khajavy and Aghaee found that only POE could predict L2 achievement directly or influence L2 achievement indirectly through the mediating role of personal bests [38]. Another study conducted by Zhao et al. with the participation of 169 Arabic multilingual learners from Saudi Arabia indicated that no direct link was found between grit and online L2 Chinese language achievement [39].

In addition, grit has been shown to be intertwined with other L2 variables, such as the ideal L2 self, motivation, language competence, and WTC [12, 40–43]. However, the association between grit and L2 WTC, which has been gradually considered in recent years, is reported with mixed findings in the current literature. Lan et al., for instance, found that grit mediated the relationship between L2 self and L2 WTC in a questionnaire survey among 842 undergraduates from three cities in China [43]. In a similar vein, Bensalem et al. administered a cross-cultural online survey investigating the intricate connections between personality traits and L2 WTC. The results confirmed that grit was a significant predictor of L2 WTC among Saudi and Moroccan university EFL learners [12]. Nevertheless, some empirical studies failed to detect a robust correlation between grit and L2 WTC. For instance, a study conducted by Lee among 647 Korean EFL learners from five schools reported that although POE was predictive of L2 WTC, COI was not a significant predictor among all participants [44]. Similarly, in a questionnaire study of 269 Iranian students from public schools and private institutes, Ebn-Abbasi and Nushi reported that only POE was directly linked to L2 WTC. [45]. Hence, despite these tentative research findings, the link between grit and L2 WTC merits further investigation.

### FLE as the mediator between teacher support, grit, and L2 WTC

In recent decades, the burgeoning interest in positive psychology has inspired scholars to explore learners’ positive emotions, among which FLE has been the most studied in the SLA field [21]. As pioneers of FLE researchers, Dewaele and MacIntyre deemed that FLE mainly stemmed from two potential sources: developing interpersonal relationships and making progress toward a goal [20]. Moreover, a particular language learning activity or a class session may bring about enjoyment for language learners [20]. A plethora of research has been conducted to explore the antecedents of FLE. Many learner internal variables (e.g., self-perceived foreign language proficiency, regulatory emotional self-efficacy, and grit) and learner external variables (e.g., teachers’ personality traits, teacher support, and classroom social climate) have been validated to significantly predict FLE [46–51].

The control-value theory (CVT) asserts that institutional factors (e.g., classroom social climate, teacher support, and teaching quality) are distal antecedents of achievement emotions and exert a significant influence on learners’ emotions, triggering a change in emotional experience for everyone in that particular environment [52]. The bulk of studies have confirmed such claims. In a cross-sectional and longitudinal study among 548 public school students in Portugal, Forsblom et al. found that students’ perceived teacher support in math classes was positively related to their perceived competence, positive value, and enjoyment [53]. Another study carried out by Zhang et al. among 300 music major university students in China suggested that teacher autonomy support was a positive predictor of students’ music enjoyment [54]. Similar findings were also corroborated in EFL contexts. According to Dewaele et al., teachers played a pivotal role in enhancing students’ FLE, and there was a strong link between teachers’ classroom behavior and the degree to which students enjoyed their EFL learning process [55]. Consistent with this, in a questionnaire study involving 564 Chinese undergraduates, Jiang and Dewaele discovered that teachers’ friendliness and joking in class were significantly related to learners’ FLE [50].

Grit could be another antecedent of FLE grounded in CVT, which states that emotions are attributable to appraisals of subjective control over learning situations and outcomes as well as the subjective value of the activities and outcomes [52]. In light of this theory, grit will also probably boost positive emotions, such as FLE, by supporting control and value appraisals. For instance, Wang and Ren stated that both POE and COI were significant predictors of FLE after conducting a survey with 171 Chinese postgraduates involved [56]. Another study run by Liu and Wang among 697 Chinese high school students indicated that grit, as a one-factor structure, could significantly positively influence FLE [48].

Additionally, prior research unveiled a positive correlation between FLE and other psychological and linguistic variables, such as foreign language classroom anxiety (FLCA), student engagement, speaking motivation and proficiency, intercultural communicative competence, and L2 WTC [12, 20, 21, 51, 57, 58].

The relationship between FLE and L2 WTC, for example, has been widely explored by scholars [58–60]. In accordance with CVT, emotions are aroused by the learning process or outcomes [52]. Supported by this theory, FLE is regarded as a positive, high-arousal, and activity-related emotion [61]. Individuals equipped with such emotions (e.g., FLE) are more likely to be activated by induced physical tension and are stimulated to give more active behavioral responses (e.g., L2 WTC). To illustrate, Feng et al. revealed the mediating role of FLE between achievement goals and WTC, as well as the effect of FLE on WTC among 408 Chinese secondary school students using a variable-centered approach. Their results indicated that a greater FLE was tightly linked to a greater WTC [59]. In an online survey of 601 Iranian EFL learners, Fathi et al. verified that FLE directly and positively predicted L2 WTC [58]. Similar findings were also evident in a questionnaire study among 328 Saudi university EFL learners conducted by Alrabai, who concluded that FLE was a significant precursor of L2 WTC [60].

It follows from the preceding that although the possible relationships between teacher support, grit, and FLE, as well as between FLE and L2 WTC, have been more or less confirmed, very few studies, to our specific knowledge, have documented how teacher support and grit influence L2 WTC with the joint effort of FLE as a mediator, thus limiting the deep understanding of the strong connection between positive institutions, positive personality traits, and positive emotions in affecting learners’ L2 WTC. Therefore, based on CVT, the current study aims to fill this gap by expanding the existing body of research and exploring the effect of teacher support and grit on L2 WTC and the mediating role of FLE.

### Hypothesis model

**Fig. 3:**
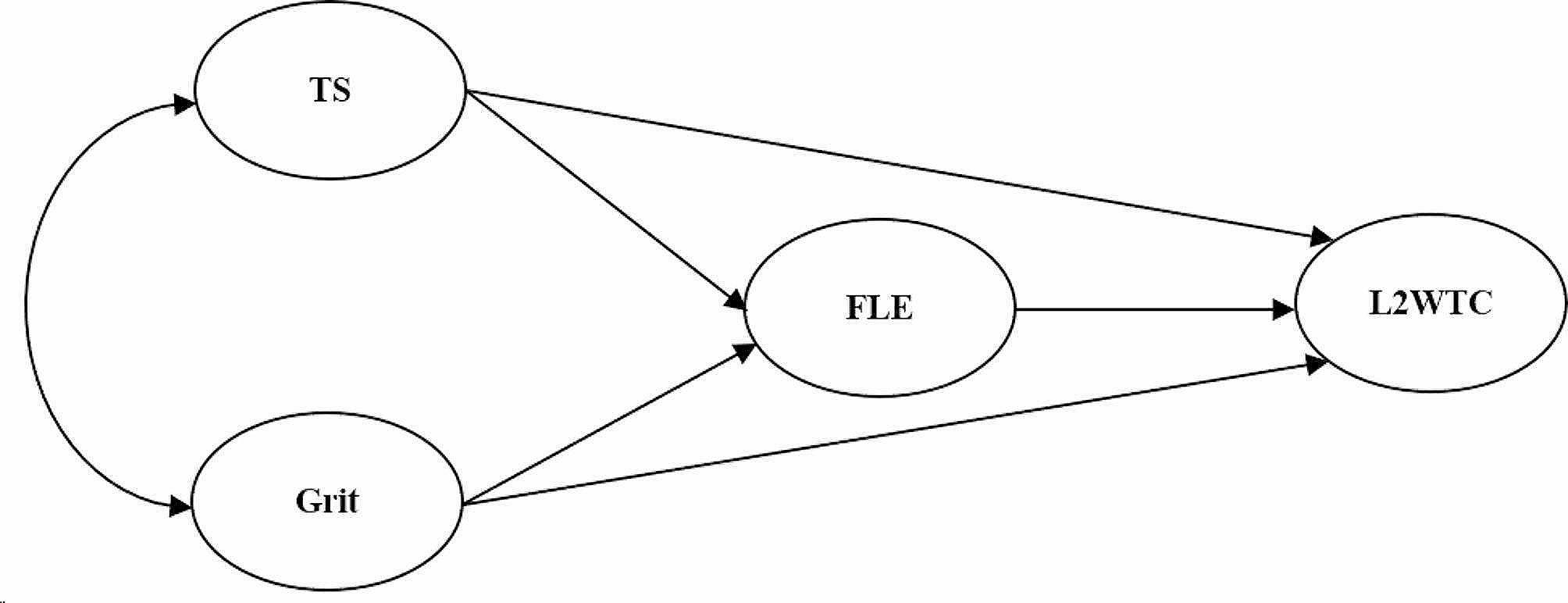
The first hypothesized model (one-factor grit). *Note* TS = teacher support, FLE = foreign language enjoyment, L2 WTC = second language willingness to communicate

**Fig. 4:**
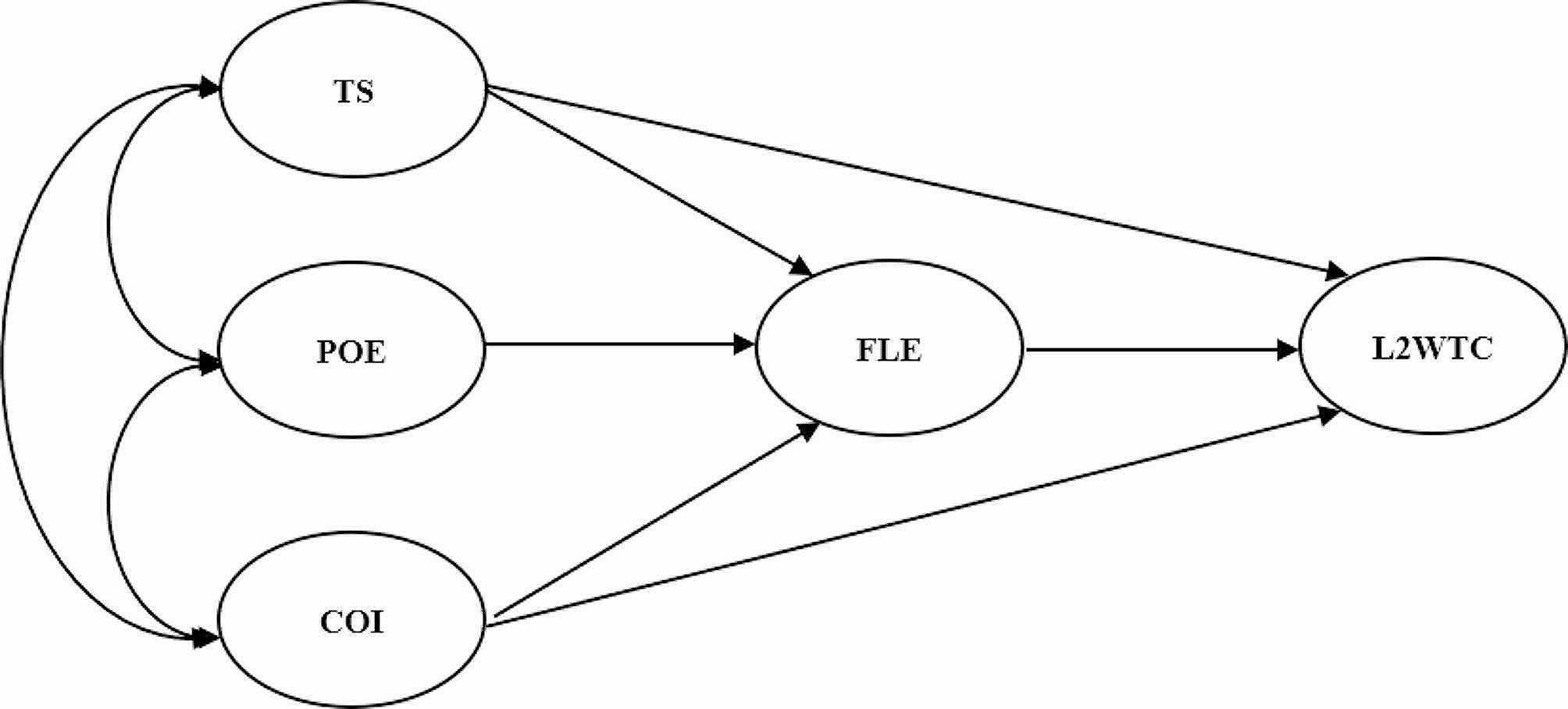
The second hypothesized model (two-factor grit)

In line with the above considerations, the current study was designed to explore two hypothesized models accordingly (see Figs. 3 and 4 for the models).

In Model 1, grit is considered a one-factor structure in which three independent factors (teacher support, grit, and FLE) may predict one dependent factor (L2 WTC) in the underlying mechanism. Three hypotheses are listed as follows.

#### Hypothesis 1

Teacher support directly and positively predicts L2 WTC.

#### Hypothesis 2

Grit directly and positively predicts L2 WTC.

#### Hypothesis 3

FLE serves as a mediator in the relationships between teacher support, grit, and L2 WTC.

Model 2 regards grit as a two-factor structure in which POE and COI are considered separately. It is designed to determine the best predictive effect of grit in L2 WTC and prevent the loss of utility of either component of grit [18]. In this model, teacher support, POE, COI, and FLE are independent factors that may significantly predict L2 WTC. Several hypotheses are listed below.

#### Hypothesis 1

Teacher support directly and positively predicts L2 WTC.

#### Hypothesis 2

POE directly and positively predicts L2 WTC.

#### Hypothesis 3

COI directly and positively predicts L2 WTC.

#### Hypothesis 4

FLE serves as a mediator in the relationships between teacher support, POE, COI, and L2 WTC.

## Methodology

### Participants

Using convenience sampling, the researchers distributed questionnaires among 624 university students from one state-owned university in China. The students were informed that the survey was only for educational purposes and that participation was voluntary. The data were collected in March 2024. After completion, we ruled out invalid questionnaires caused by regular responses and collected 619 valid surveys for an effective recovery rate of 99.2%. Of the valid samples, 255 (41.2%) were freshmen, 364 (58.8%) were sophomores, 327 (52.8%) were male, and 292 (47.2%) were female. Their mean age was 19.6 years (SD = 0.81). All the participants were native Chinese speakers with EFL learning periods of more than ten years, none of whom had ever studied or worked abroad. Before the survey, all the participants were informed of its purpose. They were also assured of the confidentiality of the information and their freedom to withdraw from the survey at any time without consequence.

### Instruments

The constructs explored in our study were measured using a composite questionnaire. The original scales for measuring students’ perceived teacher support, L2 grit, FLE, and L2 WTC were designed in English. To avoid any potential difficulty in understanding and answering the survey, we invited a PhD student majoring in English applied linguistics to translate all items in the four scales into Chinese. The Chinese version was then verified and back-translated into English by two professors in educational psychology and English applied linguistics. Furthermore, we carefully compared the English translation version with the original one, detected some linguistic errors such as spelling mistakes, word-for-word translation, or inappropriate diction, discussed them in great detail to ensure its accuracy, and agreed on the final Chinese version. All the items were assessed with a 5-point Likert scale ranging from 1 (strongly disagree) to 5 (strongly agree). The composite questionnaire was created at WJX.CN and then delivered to students via WeChat or QQ (Chinese social media platforms).

### The students’ perceived EFL teacher support scale

The scale for measuring perceived teacher support in our study originated from the Students’ Perceived EFL Teacher Support Scale synthesized by Liu and Li, who adopted items from the Child and Adolescent Social Support Scale and an open questionnaire [28, 62]. The scale consisted of three dimensions: teacher academic support (5 items, *α* = 0.897), teacher instrumental support (3 items, *α* = 0.878), and teacher emotional support (4 items, *α* = 0.889).

Two examples from the scales are: (1) “The English teacher carries out special teaching for our weak points”; (2) “The English teacher helps us choose suitable learning materials”. In this study, the scale showed high reliability (*α* = 0.922).

### L2 grit scale

In our present study, we used the L2 Grit Scale designed by Teimouri et al. to measure students’ capacity to stick to their efforts and interest in L2 learning [63]. The scale included two dimensions with good reliability indices: perseverance of effort (5 items, *α* = 0.941) and consistency of interest (4 items, *α* = 0.874). The whole scale was also tested to have excellent internal reliability (*α* = 0.896). Some sample items included (1) “I am a diligent English language learner”; (2) “I have been obsessed with learning English in the past but later lost interest.”

### Foreign language enjoyment scale

We used the Foreign Language Enjoyment Scale in our study, which was modified from the Chinese version of the Foreign Language Enjoyment Scale [64]. It included 11 items with desirable reliability (*α* = 0.884). Example items for the scale are: (1) “I have learned a lot of things in English learning”; (2) “In class, I feel proud of my English achievements”.

### L2 WTC scale

In our present study, we used the L2 WTC Scale designed by Lee and Hsieh to measure the extent to which students preferred to use English in the classroom [65]. The scale included eight items with satisfactory reliability (*α* = 0.928). Some items for the scale include: (1) “When you have a chance to make a presentation in front of a large group, you are willing to communicate in English”; (2) “When you have a chance to talk as part of a small group of strangers, you are willing to communicate in English”.

### Data analysis

We performed the data analysis with SPSS (version 27) and Amos (version 26). Initially, the Expectation Maximization algorithm was used to check for missing data, outliers, and normality. Next, we carried out a CFA test to examine the validity of the measurement instruments in Amos and evaluated the model fit indices. According to Vandenberg and Lance, the model is considered fit when *χ*^2^/*df* < 3, GFI, CFI, TLI ≥ 0.90, RMSEA ≤ 0.08, and SRMR < 0.10 [66]. Then, descriptive and correlational analyses were performed via SPSS. Finally, SEM was used to test the hypotheses listed above.

## Results

### Preliminary analysis and measurement model test

Prior to testing the measurement model, the data were screened using SPSS. The results indicated that no missing data or outliers were detected, and the absolute skewness and kurtosis values were below 2.0. Therefore, all the constructs were normally distributed according to the criteria of Kunnan and Tabachnick et al. [67, 68].

In the next step, we first performed CFA to examine the factor structure of grit (see Table 1). Based on the goodness-of-fit indices of the three models in Table 1, the first-order one-factor grit was superior to the other two potential models. Therefore, we abandoned the second hypothesized model (two-factor grit) and its subsequent four hypotheses. Grit was analyzed as a one-factor structure underlying the mechanism.

**Table 1:** Goodness-of-fit indices for the hypothesized models

Model	*χ* ^2^	*df*	CFI	TLI	RMSEA	SRMR
First-order one-factor	1262.91	290	0.920	0.910	0.041	0.074
First-order two-factor	2031.08	337	0.881	0.867	0.090	0.041
Second-order two-factor	No results can be obtained					

In addition, a CFA test were also used to check the construct validity of the latent constructs: teacher support, grit, FLE, and L2 WTC. Initially, fit indices suggested that some measurement instruments did not satisfy the recommended level of data adequacy. The factor loadings of three teacher support items, three grit items, four FLE items, and one L2 WTC item were below the threshold of 0.5. As recommended by Hair et al. [69], we modified the original model by discarding these items, and the measurement instruments showed adequate model fit (see Table 2).

**Table 2:** Measurement model of the latent constructs

	*χ* ^2^	*df*	*χ*^2^/*df*	CFI	TLI	RMSEA	Cronbach’s *α*
teacher support	50.463	21	2.403	0.984	0.973	0.051	0.922
grit	6.015	3	2.005	0.987	0.982	0.045	0.896
FLE	13.050	5	2.610	0.981	0.979	0.055	0.884
L2 WTC	12.649	7	1.807	0.978	0.977	0.042	0.928

We also evaluated the convergent and discriminant validity among the constructs. According to Fornell and Larcker, AVE values greater than 0.50 and CR values greater than 0.70 exhibited an acceptable level of convergent validity [70]. The results (see Table 3) demonstrated that all the constructs in this study met such a requirement. In addition, the correlation coefficients between the constructs were all positive and significant, showing that all the constructs were associated with each other. The square roots of the AVE values were subsequently calculated for further discriminant analysis. As a result, those values were all greater than the correlation coefficients between the constructs (see Table 3), which indicated that good discriminant validity was found between the constructs [70].

**Table 3:** Convergent and discriminant validity

Constructs	Convergent validity			Discriminant validity		
	AVE	CR	Teacher support	Grit	FLE	L2WTC
Teacher support	0.840	0.940	**0.917**			
Grit	0.653	0.904	0.647***	**0.808**		
FLE	0.559	0.884	0.671***	0.789***	**0.748**	
L2 WTC	0.682	0.927	0.469***	0.665***	0.689***	**0.826**

*Note* Figures in boldface represent the square roots of the AVE values

***p < 0.001

In a follow-up analysis, we proceeded with the descriptive and correlational analysis of the constructs (see Table 4). According to Plonsky and Oswald, the effect sizes of correlations in L2 studies can be interpreted as follows: *r* = 0.1 (small), 0.3 (medium), and 0.5 (large) [71]. Based on the results (see Table 4), teacher support was found to be strongly correlated with grit (*r* = 0.647) and FLE (*r* = 0.671) and moderately linked with L2 WTC (*r* = 0.469). Grit was identified to have a strong connection with FLE (*r* = 0.789) and L2 WTC (*r* = 0.665). FLE was determined to be strongly associated with L2 WTC (*r* = 0.689).

**Table 4:** Descriptive statistics and correlations

Constructs	Min	Max	M	SD	1	2	3	4
1. Teacher support	1.00	5.00	4.127	0.430	1.00			
2. grit	1.00	5.00	4.057	0.454	0.647***	1.00		
3. FLE	1.00	5.00	3.649	0.558	0.671***	0.789***	1.00	
4. L2 WTC	1.00	5.00	3.694	0.796	0.469***	0.665***	0.689***	1.00

### SEM results

**Fig. 5:**
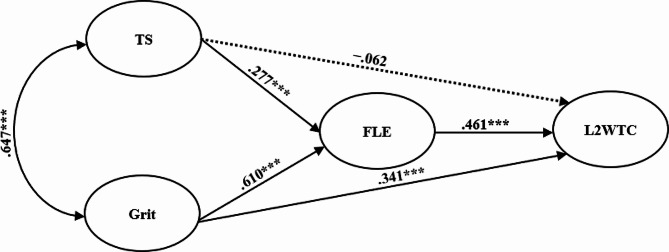
The final model. *Note* Estimates are standardized. The dotted line represents a non-significant path. ***p<0.001

Finally, SEM analysis was carried out using maximum likelihood estimation. After controlling for age, gender, and grade, the final model (see Fig. 5) revealed a good fit (*χ*^2^/*df* = 2.992, GFI = 0.907, CFI = 0.954, TLI = 0.947, RMSEA = 0.057, SRMR = 0.041). Furthermore, a bias-corrected bootstrap analysis with 5000 samples and 95% confidence intervals (CI) was conducted to test the mediating role of FLE in the relationships between teacher support, grit, and L2 WTC. Table 5 presents the results of the path analysis, which showed that grit could positively predict FLE (*β* = 0.610, *p* < 0.001) and L2 WTC (*β* = 0.341, *p* < 0.001). Besides, teacher support could also positively affect FLE (*β* = 0.277, *p* < 0.001). With zero included in the 95% CI, the path from teacher support to L2 WTC (*β* = −0.062, *p* > 0.05) was not significant, indicating that teacher support did not predict L2 WTC directly. Moreover, bootstrap results showed that FLE mediated the relationship between teacher support and L2 WTC (*β* = 0.128, *p*<0.001, 95% CI [0.075, 0.195]) as well as that between grit and L2 WTC (*β* = 0.281, *p* < 0.001, 95% CI [0.200, 0.367]). Overall, these results implied that FLE fully mediated the link between teacher support and L2 WTC while partially mediating the effect of grit on L2 WTC.

**Table 5:** Direct and indirect effects of 95% CI (controlling for age, gender, and grade)

Model pathways	B	Boot SE	*β*	*p*	Bias-corrected 95% CI	
					Lower bound	Upper bound
Direct effect						
Teacher support - FLE	0.319	0.056	0.277	***	0.174	0.359
Grit - FLE	0.671	0.048	0.610	***	0.532	0.691
FLE - L2 WTC	0.508	0.080	0.461	***	0.332	0.593
Teacher support - L2 WTC	−0.079	0.058	-0.062	0.207	−0.156	0.036
Grit - L2 WTC	0.414	0.074	0.341	***	0.213	0.457
Indirect effect						
Teacher support - FLE - L2 WTC	0.162	0.036	0.128	***	0.075	0.195
Grit - FLE - L2 WTC	0.341	0.051	0.281	***	0.200	0.367

*Note* “Bias-corrected 95% CI” pertains to the confidence intervals for the Standardized Estimate Values

***p < 0.001

## Discussion

Motivated by the theoretical framework of positive psychology and relevant empirical findings, the current study investigated how teacher support and grit predicted L2 WTC among Chinese EFL learners and whether FLE served as mediators of these associations. It underlines the weight of positive institutions, positive personality traits, and positive subjective experience in affecting learners’ L2 WTC and mirrors the recent call in SLA research to consider learner affective and emotional variables [20, 72] amid the backdrop of the growing recognition of positive psychology application in the L2 field. Some tentative findings were generated concerning the proposed model and hypotheses for the study.

First, contrary to previous studies [16, 35], our results showed that teacher support was not a significant positive predictor of L2 WTC, rejecting Hypothesis 1. The inconsistency of the research results may be caused by social-cultural and environmental factors. It is noted that sometimes learners are still unwilling to speak up or participate in a classroom discussion even if teachers support them instrumentally and emotionally because the cultural norms of many countries focus on respecting authority. As can be seen in our case, Chinese students have been taught to show respect for teachers and school stakeholders ever since they were in kindergarten. Speaking up in class without permission is regarded as an offense or severe violation of discipline, especially for children studying in elementary school. They may even receive punishment from teachers under such circumstances. In addition, although the Ministry of Education in China has launched multiple rounds of teaching reforms in recent decades, aiming to improve students’ L2 communicative abilities, English is still considered a subject throughout their studies. Since 1978, students have had to pass a higher education examination paper test (gaokao) to be admitted to universities. The test contains exercises such as listening, reading comprehension, Chinese-English translation, and writing, except for only a few coastal provinces that include oral English in the scope of the test. Therefore, it is not unusual to note that some Chinese students remain silent in class despite the tremendous efforts exerted by their EFL teachers, who are often equipped with international vision and open-mindedness.

Second, the quantitative analysis confirmed a statistically positive effect of grit on L2 WTC, which echoed the findings of Lan et al. and Bensalem et al. [12, 43], supporting Hypothesis 2. This finding also corroborates the effectiveness of the multilayered pyramid model, which states that a fixed personality feature might determine speakers’ willingness to initiate a conversation [6]. Specifically, grittier learners are inclined to maintain perseverance in their efforts and consistency of interest in the process of L2 learning despite previous setbacks and frustrations. On the one hand, L2 learners with higher levels of grit tend to persevere and work unwearyingly toward completing demanding tasks, which may help them make further progress, reflecting Duckworth et al.’s findings that gritty learners are also tenacious in accomplishing tasks and they keep them up until the last minute [36]. Concerning the positive influence of grit on learners’ L2 WTC, it can be inferred that gritty individuals are enthusiastic about finishing challenging tasks (e.g., oral communication), and they may seize every possible opportunity to start conversations with their teachers and peers. As a result, they have a greater tendency to use L2 for social interaction. On the other hand, gritty learners also exhibit strong interest and curiosity in the process of learning, propelling them to complete tough missions. This interpretation accords with Vansteenkiste et al. and Pae’s discoveries about learners’ intrinsic motivation, intrinsic goals, and learning outcomes [73, 74]. According to them, learners’ intrinsic motivation, such as having an interest in L2, is a strong determinant in building learning confidence, and those who possess intrinsic goals while completing learning activities are more dedicated and engaged [73, 74]. It can be postulated that EFL gritty learners have a burning passion for L2 and are more likely to maintain their interest in the course of learning with sustained effort. Therefore, they will do their utmost to become competent L2 speakers.

Third, SEM analysis also suggested that FLE was a significant predictor of L2 WTC, aligning with previous findings [22, 44, 58–60]. This result upholds the hypothesis proposed by Dewaele et al., who asserted that a high level of enjoyment when learning a target language was an essential precondition for learners to show their WTC in L2 [55]. It serves as another empirical affirmation to strengthen CVT, which posits that enjoyment, as a positive and activating emotion, can stimulate learners to pursue learning activities, enabling them to be more willingly engaged in L2 learning and communication [75]. The contribution of FLE to L2 WTC can also be attributed to the broaden-and-build theory put forward by Fredrickson, who stated that positive emotions broadened people’s thought-action repertoires and affected their mode of behavior as well as L2 learning outcomes [76].

Fourth, our findings also add solid evidence to the very scant studies in the current literature regarding the positive link between teacher support and grit [77, 78]. According to previous research by Shen and Guo, a significant correlation was detected between teacher support and grit, both of which were analyzed as first-order one-factor constructs [78]. In addition, Hejazi and Sadoughi found that when a second-order three-factor teacher support and a second-order two-factor grit were considered under SEM, different dimensions of these constructs (i.e., Instrumental teacher support, Appraisal teacher support, Emotional teacher support, POE, and COI) were also significantly correlated [77]. This might be explained by the nature of grit. As a positive personality trait, grit entails learners’ perseverance and interest in undertaking a demanding task. Perseverance and interest, however, are closely tied to academic, instrumental, and emotional support from teachers [79]. Hence, it seems quite plausible to infer that the more support teachers render, the grittier the learners will be. Another explanation for this association lies in self-determination theory. By providing autonomy support, teachers can help students improve their psychological well-being with effective stress-coping strategies and further enhance their resilience with sustained effort [80].

Fifth, although teacher support could not predict L2 WTC directly, its impact on L2 WTC cannot be ignored. An indirect effect through FLE was detected in our study, suggesting that both teacher support and grit could affect L2 WTC via the mediating role of FLE. This mediating effect is a reflection of the dynamic nature of achievement emotions by CVT, as it “affects the cognitive, motivational, and regulatory processes mediating learning and achievement, as well as psychological well-being, happiness, and life satisfaction” [81]. This result also implies that the associations and interconnectedness among the Three Pillars (i.e., positive institutions, positive personality traits, and positive subjective experience), represented by teacher support, grit, and FLE in our study, contribute greatly to language development [14], indicating that the power of positive institutions and positive personality traits could be maximized through the positive effect of positive subjective experience. In other words, gritty learners or learners with much teacher support are likely to experience more enjoyment in language learning, boosting them to engage in L2 interactions more enthusiastically. Illuminated by such findings, EFL teachers may motivate learners to practice oral language diligently through a plethora of captivating activities both inside and outside the classroom. With the enhancement of their interest and FLE, they will be more inspired to speak.

### Conclusion and pedagogical implications

Based on some relevant theories of positive psychology, this study engaged 619 university students in China to examine the associations between teacher support, grit, FLE, and L2 WTC. SEM results evidenced that grit could directly and positively predict L2 WTC. In addition, FLE was found to positively mediate the link between teacher support, grit, and L2 WTC. Theoretically, this study provides empirical evidence for the applicability of CVT in the field of SLA [52, 81]. The results highlighted that the Three Pillars of positive psychology (i.e., positive institutions, positive personality traits, and positive subjective experience) would facilitate L2 communication. Considering that L2 learning and acquisition are long, demanding, and tedious processes that involve a large amount of time and dedication, learners should be fully prepared to set well-defined goals and resort to helpful external strategies to address various challenges. Besides, our findings affirm the role of teachers whose support is exceptionally vital for enhancing learners’ persistence and interest in L2 learning and promoting their FLE in the learning process, which can, in turn, ignite learners’ desire to communicate. Based on the theoretical model and findings of the current study, some pedagogical implications for EFL pedagogy can be summarized as follows.

First, given the direct and indirect effects of grit on L2 WTC, EFL teachers are recommended to cultivate this positive personality trait with various motivational strategies. For instance, the stories of numerous inspiring models whose grit propelled them to achieve success in L2 learning can be introduced to learners in detail, helping them realize that learning achievement can be obtained by continuous grittiness and encouraging them to repeat the stories or make a self-reflection in L2 orally. In addition, by assigning long-term L2 learning projects, teachers can train learners to be grittier while completing those challenging tasks. Second, our findings verified the role of FLE in strengthening L2 WTC. Thus, we may encourage EFL teachers to set attainable learning goals that cultivate learners’ communicative competence via FLE. To this end, speaking tasks in class should be prioritized in daily teaching practice to help learners realize that communication is the ultimate goal of learning a target language. In addition, teachers can prepare a wide range of engaging topics and exciting activities to allow the full participation of learners in class, such as dubbing English movies, imitating celebrity speeches, singing English songs, reciting famous poems, and debating on interesting issues. With smiles lingering on learners’ faces, their L2 WTC soars. Third, considering the strong correlation between teacher support and FLE, it is also advisable for EFL teachers to support learners academically, emotionally, and instrumentally. For example, an online chatroom in WeChat or Telegram can be created after class, through which learners can turn to their teachers for academic help whenever and wherever possible. It is suggested that teachers foster a stress-free classroom environment characterized by warmth and empathy and periodically share useful learning tools or L2 materials. This approach ensures that all learners feel supported and perceive daily progress in their studies. Finally, it is also suggested that EFL teachers join various training workshops to learn how to provide learners with different types of support and foster positive personality traits. In a nutshell, all of these combined efforts may effectively stimulate learners’ readiness for L2 communication.

### Limitations and future research directions

Although the present study contributed to the exploration of various constructs affecting L2 WTC, there are still some limitations involved in the investigation. First, cross-sectional, self-report instruments for measuring learners’ perceived teacher support, L2 grit, FLE, and L2 WTC were utilized in the study for ease of convenience, which may give rise to potential biases and influence the accuracy of the research results. Second, the sample size of the study was relatively small and restricted to a specific group of students in one country. The research findings, therefore, might not be generalizable to other EFL contexts. Future studies ought to consider a longitudinal design with qualitative and mixed methods approaches to trace the dynamic interactions of various constructs. Moreover, larger samples of EFL learners from different countries and cultural backgrounds are needed for future research endeavors. As mentioned in the recent literature by Henry and MacIntyre, “WTC develops over a timescale that is best measured in weeks and months, that communication behavior needs to be investigated in the context of community-based speech events (as a means of complementing work in labs and classrooms)” [27]. With those months-long timescales and narrative methods, participants’ processes of growth and language learning experience can be more clearly unfolded [27]. Third, the current study only considered FLE as a positive emotion. However, some negative emotions (e.g., FLCA and foreign language boredom) are closely related to FLE, and sometimes, learners can experience both positive and negative emotions simultaneously [20]. Therefore, a holistic approach is necessary for future research under the existing mechanism, and other emotions should also be explored for further discussion.

## Data Availability

The data is available from the authors upon reasonable request.

## References

[1] Murray N, Liddicoat AJ, Zhen G, Mosavian P (2023). Constraints on innovation in English language teaching in hinterland regions of China. Lang Teach Res.

[2] Ngoc BM, Barrot JS (2023). Current landscape of English language teaching research in Southeast Asia: a bibliometric analysis. Asia-Pacific Educ Researcher.

[3] MacIntyre PD (2020). Expanding the theoretical base for the dynamics of willingness to communicate. Stud Second Lang Learn Teach.

[4] Eisenchlas SA (2009). Conceptualizing ‘communication’in second language acquisition. Australian J Linguistics.

[5] MacIntyre PD, Doucette J (2010). Willingness to communicate and action control. System.

[6] MacIntyre PD, Clément R, Dörnyei Z, Noels KA (1998). Conceptualizing willingness to communicate in a L2: a situational model of L2 confidence and affiliation. Mod Lang J.

[7] Dewaele JM, Pavelescu LM (2021). The relationship between incommensurable emotions and willingness to communicate in English as a foreign language: a multiple case study. Innov Lang Learn Teach.

[8] Pavelescu LM. Emotion, motivation and willingness to communicate in the language learning experience: a comparative case study of two adult ESOL learners. Lang Teach Res. 2023;0(0). 10.1177/13621688221146884.

[9] MacIntyre PD (2007). Willingness to communicate in the second language: understanding the decision to speak as a volitional process. Mod Lang J.

[10] Munezane Y (2015). Enhancing willingness to communicate: relative effects of visualization and goal setting. Mod Lang J.

[11] Zarrinabadi N (2014). Communicating in a second language: investigating the effect of teacher on learners’ willingness to communicate. System.

[12] Bensalem E, Thompson AS, Alenazi F. The role of grit and enjoyment in EFL learners’ willingness to communicate in Saudi Arabia and Morocco: a cross-cultural study. J Multiling Multicultural Dev. 2023;1–16. 10.1080/01434632.2023.2200750.

[13] Seligman ME, Csikszentmihalyi M. Positive psychology: an introduction. Volume 55. American Psychological Association; 2000. p. 5. 1. 10.1037//0003-066x.55.1.5 11392865

[14] MacIntyre PD. So far so good: an overview of positive psychology and its contributions to SLA. Springer International Publishing; 2016. pp. 3–20.

[15] Sadoughi M, Hejazi SY (2021). Teacher support and academic engagement among EFL learners: the role of positive academic emotions. Stud Educational Evaluation.

[16] Hejazi SY, Sadoughi M, Peng JE (2023). The structural relationship between teacher support and willingness to Communicate: the mediation of L2 anxiety and the moderation of Growth Language Mindset. J Psycholinguist Res.

[17] Shirvan ME, Alamer A. Modeling the interplay of EFL learners’ basic psychological needs, grit and L2 achievement. J Multiling Multicultural Dev. 2022;1–17. 10.1080/01434632.2022.2075002.

[18] Zhao X, Wang D (2023). Grit, emotions, and their effects on ethnic minority students’ English language learning achievements: a structural equation modelling analysis. System.

[19] Zhao X, Wang D (2023). Grit in second language acquisition: a systematic review from 2017 to 2022. Front Psychol.

[20] Dewaele JM, MacIntyre PD (2014). The two faces of Janus? Anxiety and enjoyment in the foreign language classroom. Stud Second Lang Learn Teach.

[21] Fattahi N, Ebn-Abbasi F, Botes E, Nushi M. Nothing ventured, nothing gained: the impact of enjoyment and boredom on willingness to communicate in online foreign language classrooms. Lang Teach Res. 2023;0(0). 10.1177/13621688231194286.

[22] Li C, Dewaele JM, Pawlak M, Kruk M (2022). Classroom environment and willingness to communicate in English: the mediating role of emotions experienced by university students in China. Lang Teach Res.

[23] Chen YL, Cheng HF, Tang HW, Wang C (2021). The roles of English varieties and L2 motivation in English learners’ willingness to communicate in the internationalization at home (IaH) context. Int Rev Appl Linguist Lang Teach.

[24] Oz H (2014). Big five personality traits and willingness to communicate among foreign language learners in Turkey. Social Behav Personality: Int J.

[25] Zhou L, Xi Y, Lochtman K (2023). The relationship between second language competence and willingness to communicate: the moderating effect of foreign language anxiety. J Multiling Multicultural Dev.

[26] Jaramillo Cherrez N, Nadolny L (2023). Willingness to communicate and oral communicative performance through asynchronous video discussions. Lang Learn Technol.

[27] Henry A, MacIntyre PD. Willingness to communicate, multilingualism and interactions in community contexts. Volume 22. Channel View; 2023.

[28] Liu H, Li X (2023). Unravelling students’ perceived EFL teacher support. System.

[29] Granziera H, Liem GAD, Chong WH, Martin AJ, Collie RJ, Bishop M, Tynan L (2022). The role of teachers’ instrumental and emotional support in students’ academic buoyancy, engagement, and academic skills: a study of high school and elementary school students in different national contexts. Learn Instruction.

[30] Patrick H, Ryan AM (2005). Identifying adaptive classrooms: dimensions of the classroom social environment. What do children need to flourish? Conceptualizing and measuring indicators of positive development.

[31] Chiu TK, Moorhouse BL, Chai CS, Ismailov M. Teacher support and student motivation to learn with Artificial Intelligence (AI) based chatbot. Interact Learn Environ. 2023;1–17. 10.1080/10494820.2023.2172044.

[32] Zhang H, Sun C, Liu X, Gong S, Yu Q, Zhou Z (2020). Boys benefit more from teacher support: effects of perceived teacher support on primary students’ creative thinking. Think Skills Creativity.

[33] Brock LL, Curby TW (2014). Emotional support consistency and teacher–child relationships forecast social competence and problem behaviors in prekindergarten and kindergarten. Early Educ Dev.

[34] Tao Y, Meng Y, Gao Z, Yang X (2022). Perceived teacher support, student engagement, and academic achievement: a meta-analysis. Educational Psychol.

[35] Peng JE, Woodrow L (2010). Willingness to communicate in English: a model in the Chinese EFL classroom context. Lang Learn.

[36] Duckworth AL, Peterson C, Matthews MD, Kelly DR (2007). Grit: perseverance and passion for long-term goals. J Personal Soc Psychol.

[37] MacIntyre P, Khajavy GH (2021). Grit in second language learning and teaching: introduction to the special issue. J Psychol Lang Learn.

[38] Khajavy GH, Aghaee E. The contribution of grit, emotions and personal bests to foreign language learning. J Multiling Multicultural Dev. 2022;1–15. 10.1080/01434632.2022.2047192.

[39] Zhao X, Sun PP, Gong M. The merit of grit and emotions in L2 Chinese online language achievement: a case of arabian students. Int J Multiling. 2023;1–27. 10.1080/14790718.2023.2202403.

[40] Sun W, Shi H, Yan Y. Contributions of Ideal L2 self, grit, and Boredom to Engagement in an EFL context: a structural equation modeling Approach. Asia-Pacific Educ Researcher. 2023;1–12. 10.1007/s40299-023-00786-2.

[41] Chen X, Lake J, Padilla AM (2021). Grit and motivation for learning English among Japanese university students. System.

[42] Alamer A (2021). Grit and language learning: construct validation of L2-Grit scale and its relation to later vocabulary knowledge. Educational Psychol.

[43] Lan G, Nikitina L, Woo WS (2021). Ideal L2 self and willingness to communicate: a moderated mediation model of shyness and grit. System.

[44] Lee JS (2022). The role of grit and classroom enjoyment in EFL learners’ willingness to communicate. J Multiling Multicultural Dev.

[45] Ebn-Abbasi F, Nushi M (2022). EFL learners’ grit, classroom enjoyment and their willingness to communicate: Iranian public school versus private English language institute learners. Asian-Pacific J Second Foreign Lang Educ.

[46] Li C. Foreign language learning boredom and enjoyment: the effects of learner variables and teacher variables. Lang Teach Res. 2022;0(0). 10.1177/13621688221090324.

[47] Pan X, Yuan Z (2023). Examining the association between peer support and English enjoyment in Chinese university students: the mediating role of regulatory emotional self-efficacy. Front Psychol.

[48] Liu E, Wang J (2021). Examining the relationship between grit and foreign language performance: enjoyment and anxiety as mediators. Front Psychol.

[49] Ahmadi-Azad S, Asadollahfam H, Zoghi M (2020). Effects of teacher’s personality traits on EFL learners’ foreign language enjoyment. System.

[50] Jiang Y, Dewaele JM (2019). How unique is the foreign language classroom enjoyment and anxiety of Chinese EFL learners?. System.

[51] Mohammad Hosseini H, Fathi J, Derakhshesh A, Mehraein S (2022). A model of classroom social climate, foreign language enjoyment, and student engagement among English as a foreign language learners. Front Psychol.

[52] Pekrun R (2006). The control-value theory of achievement emotions: assumptions, corollaries, and implications for educational research and practice. Educational Psychol Rev.

[53] Forsblom L, Peixoto F, Mata L (2021). Perceived classroom support: longitudinal effects on students’ achievement emotions. Learn Individual Differences.

[54] Zhang YH, Zhao YH, Luo YY, Yang X, Tan D (2022). The relation between autonomy support and music enjoyment in online learning for music undergraduates in the post-COVID-19 era. Front Psychol.

[55] Dewaele JM, Witney J, Saito K, Dewaele L (2018). Foreign language enjoyment and anxiety: the effect of teacher and learner variables. Lang Teach Res.

[56] Wang Y, Ren W (2024). L2 grit and pragmatic comprehension among Chinese postgraduate learners: enjoyment and anxiety as mediators. Int J Appl Linguistics.

[57] Tsang A, Lee JS (2023). The making of proficient young FL speakers: the role of emotions, speaking motivation, and spoken input beyond the classroom. System.

[58] Fathi J, Pawlak M, Mehraein S, Hosseini HM, Derakhshesh A (2023). Foreign language enjoyment, ideal L2 self, and intercultural communicative competence as predictors of willingness to communicate among EFL learners. System.

[59] Feng E, Wang Y, King RB. Achievement goals, emotions and willingness to communicate in EFL learning: combining variable-and person-centered approaches. Lang Teach Res. 2023;13621688221146887. 10.1177/13621688221146887.

[60] Alrabai F. Modeling the relationship between classroom emotions, motivation, and learner willingness to communicate in EFL: applying a holistic approach of positive psychology in SLA research. J Multiling Multicultural Dev. 2022;1–19. 10.1080/01434632.2022.2053138.

[61] Pekrun R, Goetz T, Daniels LM, Stupnisky RH, Perry RP (2010). Boredom in achievement settings: exploring control–value antecedents and performance outcomes of a neglected emotion. J Educ Psychol.

[62] Malecki CK, Demaray MK (2003). What type of support do they need? Investigating student adjustment as related to emotional, informational, appraisal, and instrumental support. School Psychol Q.

[63] Teimouri Y, Plonsky L, Tabandeh F (2022). L2 grit: passion and perseverance for second-language learning. Lang Teach Res.

[64] Li C, Jiang G, Dewaele JM (2018). Understanding Chinese high school students’ foreign language enjoyment: validation of the Chinese version of the foreign language enjoyment scale. System.

[65] Lee JS, Hsieh JC (2019). Affective variables and willingness to communicate of EFL learners in in-class, out-of-class, and digital contexts. System.

[66] Vandenberg RJ, Lance CE (2000). A review and synthesis of the measurement invariance literature: suggestions, practices, and recommendations for organizational research. Organizational Res Methods.

[67] Kunnan AJ (1998). An introduction to structural equation modelling for language assessment research. Lang Test.

[68] Tabachnick BG, Fidell LS, Ullman JB (2013). Using multivariate statistics.

[69] Hair JF Jr., Black WC, Babin BJ, Anderson RE. Multivariate data analysis. 7th ed. Pearson Education Limited; 2014.

[70] Fornell C, Larcker DF (1981). Evaluating structural equation models with unobservable variables and measurement error. J Mark Res.

[71] Plonsky L, Oswald FL (2014). How big is big? Interpreting effect sizes in L2 research. Lang Learn.

[72] MacIntyre PD, Wang L (2021). Willingness to communicate in the L2 about meaningful photos: application of the pyramid model of WTC. Lang Teach Res.

[73] Vansteenkiste M, Simons J, Lens W, Sheldon KM, Deci EL (2004). Motivating learning, performance, and persistence: the synergistic effects of intrinsic goal contents and autonomy-supportive contexts. J Personal Soc Psychol.

[74] Pae TI (2008). Second language orientation and self-determination theory: a structural analysis of the factors affecting second language achievement. J Lang Social Psychol.

[75] Pekrun R, Perry RP. Control-value theory of achievement emotions. International handbook of emotions in education. Routledge; 2014. pp. 120–41.

[76] Fredrickson BL (2004). The broaden–and–build theory of positive emotions. Philosophical Trans Royal Soc Lond Ser B: Biol Sci.

[77] Hejazi SY, Sadoughi M (2023). How does teacher support contribute to learners’ grit? The role of learning enjoyment. Innov Lang Learn Teach.

[78] Shen Y, Guo H (2022). Increasing Chinese EFL learners’ grit: the role of teacher respect and support. Front Psychol.

[79] Liu H, Li X, Yan Y. Demystifying the predictive role of students’ perceived foreign language teacher support in foreign language anxiety: the mediation of L2 grit. J Multiling Multicultural Dev. 2023;1–14. 10.1080/01434632.2023.2223171.

[80] Weinstein N, Ryan RM (2011). A self-determination theory approach to understanding stress incursion and responses. Stress Health.

[81] Pekrun R, Elliot AJ, Maier MA (2006). Achievement goals and discrete achievement emotions: a theoretical model and prospective test. J Educ Psychol.

